# Immune checkpoint inhibitors plus neoadjuvant chemotherapy in early triple-negative breast cancer: a systematic review and meta-analysis

**DOI:** 10.1186/s12885-021-08997-w

**Published:** 2021-11-23

**Authors:** Yuanfang Xin, Guoshuang Shen, Yonghui Zheng, Yumei Guan, Xingfa Huo, Jinming Li, Dengfeng Ren, Fuxing Zhao, Zhen Liu, Zitao Li, Jiuda Zhao

**Affiliations:** Breast Disease Diagnosis and Treatment Center of Affiliated Hospital of Qinghai University & Affiliated Cancer Hospital of Qinghai University, Xining, 810000 China

**Keywords:** PD-1/PD-L1 inhibitors, Neoadjuvant chemotherapy, Triple-negative breast cancer

## Abstract

**Purpose:**

Some studies have shown that Immune checkpoint inhibitors (ICIs) have a favorable efficacy in advanced triple negative breast cancer (TNBC) patients, but the results are controversial in neoadjuvant chemotherapy (NACT) stage. The purpose of this study is to evaluate the efficacy and safety after NACT plus ICIs in early TNBC patients.

**Methods:**

After searching PubMed, EMBASE, the Cochrane library and several mainly oncology conferences up to 30 January 2021 systematically, and define randomized controlled trials (RCTs) exploring the efficacy and safety of programmed death protein-1/programmed cell death-Ligand 1(PD-1/PD-L1) inhibitors plus neoadjuvant chemotherapy in TNBC patients. The primary endpoint was the pathological complete response (pCR) in intention-to-treat populations (ITT), and the secondary endpoints were event-free survival (EFS) and safety analysis in the ITT populations.

**Results:**

Six RCTs (*N* = 2142) were included in our meta-analysis; NACT plus ICIs increased pCR rates compared with NACT in intention-to-treat (ITT) populations (OR: 1.91; 95% CI: 1.32–2.78, *P* < 0.001). The pCR rate also increased in both PD-L1 positive (OR: 1.65; 95% CI: 1.26–2.16, *P* < 0.001) and PD-L1 negative patients (OR: 1.56; 95% CI: 1.04–2.33, *P* = 0.03), especially in PD-L1 positive patients. The benefit was also observed in nodal-positive populations (OR: 2.52; 95% CI: 1.69–3.77, *P* < 0.001) and Eastern Cooperative Oncology Group performance-status score (ECOG PS) 0 subgroup (OR: 1.90; 95% CI: 1.42–2.53, *P* < 0.001). Three RCTs (*N* = 1615) reported EFS and the results showed that adding PD-1/PD-L1 inhibitors increased EFS (HR 0.65, 95% CI 0.50–0.83, *P* = 0.0007) in ITT populations with a short follow-up time. In the safety analysis of 2205 patients with early TNBC from five eligible studies, NACT plus ICIs had a higher risk of grade 3–4 diarrhea (OR: 2.54; 95% CI: 1.21–5.32; *P* = 0.01), any grade of adverse effects(AEs)including vomiting (OR: 1.37; 95% CI: 1.00–1.86; *P* = 0.05), hyperthyroidism (OR: 6.04; 95% CI: 2.39–15.29; *P* < 0.001), and hypothyroidism (OR: 5.04; 95% CI: 3.02–8.39; *P* < 0.001).

**Conclusions:**

PD-1/PD-L1 inhibitors combined with chemotherapy can improve pCR rates and EFS, and with an increased incidence of some immune-related AEs compared with chemotherapy alone. NACT plus ICIs might be an option in patients with in PD-L1 positive and high-risk populations with positive nodal disease early TNBC.

**Supplementary Information:**

The online version contains supplementary material available at 10.1186/s12885-021-08997-w.

## Introduction

Triple-negative breast cancer (TNBC) is the breast cancer subtype with the worst prognosis due to its invasiveness and high recurrence and metastasis rates [1]. Therefore, neoadjuvant treatment is particularly important in patients with early TNBC because it could downsize the stage, increase the chance of surgery or breast-conserving surgery, test the sensitivity of patients to chemotherapy, and assess prognosis according to the pathological response [2, 3].

TNBC has few therapeutic targets, and it is not sensitive to endocrine drugs, making it difficult for targeted and endocrine therapies to be used for its treatment. Treatment mainly relies on chemotherapy, but the curative effect is poor [1, 4]. In recent years, many drugs are available for the treatment of patients with TNBC, such as immunotherapy, poly ADP-ribose polymerase (PARP) inhibitors [5, 6], and other targeted therapies [4, 7, 8]. Bevacizumab can be used for the treatment of metastatic triple negative breast cancer in certain circumstances. PARP inhibitors are mainly used for breast cancer patients with germline BRCA mutation [9, 10]. Platinum and PARP inhibitors could improve pathological complete response (pCR), but platinum drugs could increase serious toxicity [11, 12].

Due to characteristics such as large tumor mutation load, high expression of programmed cell death-Ligand 1(PD-L1), and high proportion of tumor-infiltrating lymphocytes, immunotherapy might be effective in patients with TNBC [13, 14]. The results of IMpassion130 and Keynote-355 suggested that immune checkpoint inhibitors (ICIs) plus chemotherapy are effective in patients with advanced TNBC and predicted that it would yield promising results in patients with early TNBC [15, 16]. Based on the research prospects, a number of studies on neoadjuvant chemotherapy (NACT) plus ICIs in patients with early TNBC are ongoing. Although FDA has approved pembrolizumab in combination with neo-adjuvant chemotherapy for neoadjuvant therapy of early TNBC patients based on the results of Keynote-522 trial, the results of similar studies have been inconsistent, so it is necessary to conduct meta-analysis of these studies [17–23]. Therefore, our meta-analysis aimed to further confirm the efficacy of NACT plus immunotherapy.

## Methods

### Literature search methods

A comprehensive literature search of PubMed, EMBASE, the Cochrane library, European Society for Medical Oncology (ESMO) Meeting, American Society of Clinical Oncology (ASCO) Meeting, and San Antonio Breast Cancer Symposium (SABCS) was conducted, the studies that conform to the standard were selected, the date was up to January 30, 2021, and the language was limited to English. For other relevant studies, a systematic hand search was conducted, and the abstracts and full texts were carefully read for screening. A total of 2142 patients from six randomized controlled trials (RCTs) were included in this meta-analysis [17–23]. The keywords used in the search were “immunotherapy” or “immune check-point inhibitors” or “programmed death protein-1(PD-1)” or “PD-L1” or “Pembrolizumab” or “Atezolizumab” or “Durvalumab” or “neoadjuvant chemotherapy” or “preoperative” and “breast cancer” or “triple negative breast cancer” or “TNBC” or “breast tumor” or “breast neoplasm.” Two authors (YX and YZ) independently conducted the systematic literature search and extracted the data. Then the results are summarized, and the disagreement was handled through discussion, unresolved disagreements are mainly referred to suggestions from a third party. This meta-analysis was based on the Preferred Reporting Items for Systematic Reviews and Meta-analyses (PRISMA) reporting guidelines [24].

### Study selection criteria

Studies were eligible if they met the following inclusion criteria: (1) phase II or III RCTs and eligible patients were patients with early TNBC confirmed by immunohistochemistry; (2) treatment with neoadjuvant chemotherapy plus ICIs in the experimental arm and placebo plus chemotherapy in the control arm; (3) primary endpoint was pCR, defined as ypT0/Tis ypN0 or ypT0 ypN0 or ypT0/Tis, and available data on pCR in the experimental group and control group. The excluded studies were (1) non-RCTs, cohort studies, retrospective studies, or meta-analyses; patients had other subtypes of breast cancer; (2) the date for the studies was incomplete; and (3) studies on advanced breast cancer and patients who received adjuvant therapy.

### Date extraction

The following data were collected from each study: study name, author name, publication time, journal, study type, study phase, chemotherapy regimen, the total number of patients, median age, median follow-up, the number of patients achieving pCR and pCR with different expression states of PD-L1 in the study and control groups, the number of patients achieving event-free survival (EFS), grade 3–4 adverse events (AEs) rate, and any grade of AEs in the two groups after treatment, including anemia, neutropenia, nausea, alopecia, fatigue, diarrhea, vomiting, asthenia, rash, peripheral neuropathy, infusion reaction and severe skin reaction, hyperthyroidism, and hypothyroidism.

### Study objectives

The primary aim of this meta-analysis was to compare the pCR rates of NACT plus ICIs versus NACT in patients with early TNBC. Subgroups were created according to the different expression status of PD-L1, nodal status, and Eastern Cooperative Oncology Group performance-status score (ECOG PS). The secondary aim was to determine the EFS and safety in intention-to-treat populations (ITTs). Finally, we evaluated the incidence of any grade and grade 3–4 AEs after receiving NACT plus ICIs and NACT.

### Statistical analysis

The Review Manager 5.3 software (Cochrane Collaboration) was used to create a forest plot and funnel plot, and heterogeneity was analyzed using the Q value statistic and I^2^ value statistic tests; *P* > 0.1 indicated that there was no heterogeneity, while *P* < 0.1 confirmed that there was heterogeneity among the studies, when I^2^ was larger, the heterogeneity was higher. When there was obvious heterogeneity between studies (I^2^ > 50%, *P* < 0.1), the random effects model was adopted; otherwise, the fixed effect model was adopted (I^2^ < 50%, *P* > 0.1). Odds ratios (ORs) were used to analyze pCR, subgroups analysis and adverse events (AEs) and hazard ratios (HRs) with 95% confidence intervals (CI) were used to analyze EFS. Statistical significance was defined as a *p*-value < 0.05.

### Quality assessment

The Cochrane quality assessment tool was used to evaluate the quality of each included study and the risk of bias from six fields, and categories including “low bias,” “unclear,” and “high bias” were used to determine each indicator. A funnel plot was used to evaluate the publication bias (eFigures 3 and 4 in the supplement).

## Results

**Fig. 1:**
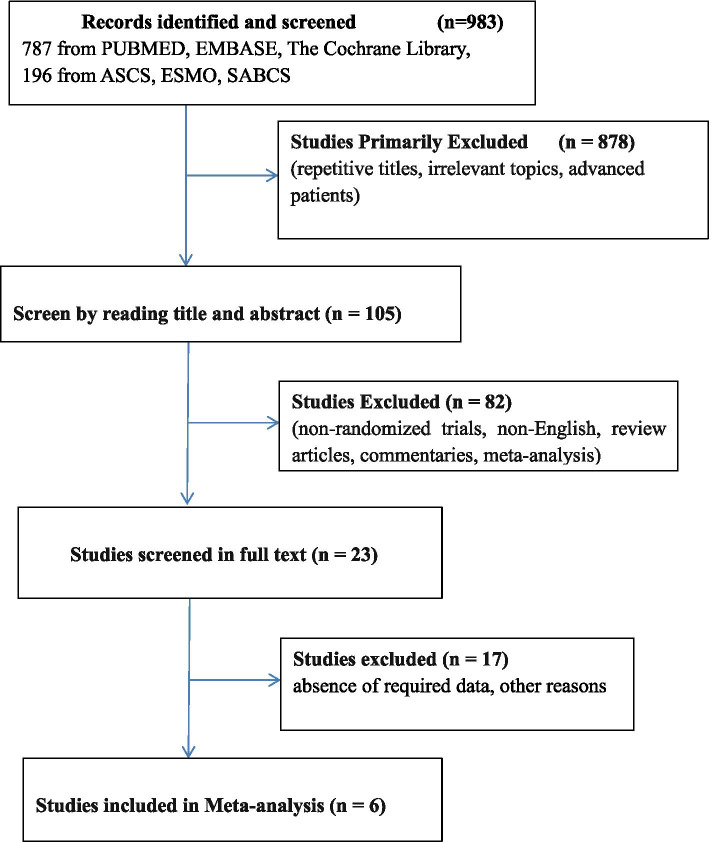
The PRISMA flow chart summarizing the process for the identification of eligible randomized controlled trials

Through comprehensive database retrieval, a total of 983 research records were determined. According to the inclusion and exclusion criteria, six studies with 2142 patients were included in this meta-analysis; four studies had full text [17–20], and two studies were published in the form of abstracts (NeoTRIPaPDL1 and Nci 10,013) [21, 22] (Fig. 1).

A total of six RCTs were included in this meta-analysis, and the characteristics of each study are shown in Table 1. Regarding the choice of neoadjuvant treatment regimen, two studies (KEYNOTE-522 and I-SPY2 trial) used pembrolizumab as the immunotherapy agent [17, 18], three RCTs (NeoTRIPaPDL1, IMpassion031, and Nci 10,013, respectively) [20–22] used atezolizumab, and only one RCT used durvalumab [19].Five RCTs used an anthracycline-and taxane-based NACT (with or without a platinum agent) [17–20, 22], and one RCT (NeoTRIPaPDL1) used anthracycline-free chemotherapy backbones [21].

**Table 1:** Characteristics of 6 trials included in this meta-analysis

Source	Year	Journal	Phase	Treatment	No. of patients	Median age	Primary endpoints	pCR in ITT(%)
KEYNOTE-522, Schmid et al. [17]	2020	New England Journal of Medicine	3	E:Pembro+pac + Cb → AC C:pbo + pac + Cb → AC	784 390	49(22–80) 48(24–79)	pCR and EFS in ITT	64.8(260/401) 51.2(103/201)
GeparNuevo study, Loibl et al. [19]	2019	Annals of Oncology	2	E:Durva+nab-pac → EC C:Pbo + nab-pac → EC	88 86	49.5(23–76)	pCR in ITT	53.4(47/88) 44.2(38/86)
NeoTRIPaPDL1, Gianni et al. [21]	2019	Cancer Research	3	E:Atezo+Cb + nab-pac C: Cb + nab-pac	138 142	50	EFS	43.5(60/138) 40.8(58/142)
I-SPY2 Trial, Nanda et al. [18]	2020	JAMA oncology	2	E:Pembro+pac → AC C:Pbo + pac → AC	29 85	50(27–71) 47(24–77)	pCR in ITT	60(17/28) 22(17/79)
Impassion031, Elizabethet al. [20]	2020	The Lancet	3	E:Atezo+nab-pac → AC C:Pbo + nab-pac → AC	165 168	51(22–76) 51(26–78)	pCR in ITT and in PD-L1+ populations	57.6(95/165) 41.1(69/168)
Nci 10,013, Foluso et al. [22]	2021	Cancer Research	2	E:Atezo+pac + Cb C:Pac + Cb	45 22	52(25–78)	pCR and TIL percentages in ITT	55.6(25/45) 18.8(3/16)

*E* Experimental, *C* Control, *pCR* pathological complete response, *EFS* event-free survival, *ITT* intention-to-treat populations, *PD-L1+* programmed death ligand 1 positive

A total of 2142 patients with early TNBC were included in our meta-analysis, 1249 patients received PD-1/PD-L1 inhibitors plus chemotherapy, and 893 patients received chemotherapy alone. Furthermore, two RCTs [18, 22] (I-SPY2 and Nci 10,013) did not distinguish PD-L1 expression status, and two trials reported pCR results based on the nodal status and ECOG PS [17, 20]. The median age in the study and control groups was 49.5 years (range,47–51 years), and the median follow-up period was 20.2 months (range, 15–34 months).

### Pathological complete response rates

**Fig. 2:**
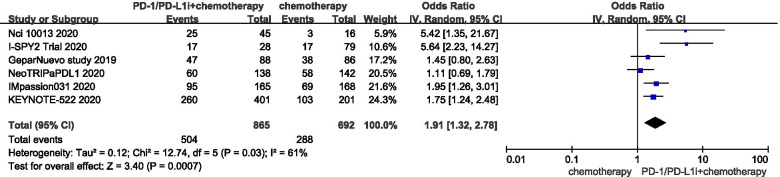
Pooled odds ratios to access pathological complete response of neoadjuvant chemotherapy plus immune checkpoint inhibitors versus neoadjuvant chemotherapy in the overall populations

A total of six RCTs with 2142 patients were included in this meta-analysis [17, 22], the pooled analysis of pCR was performed only on 1557 patients, the remaining 585 patients were not performed to pCR analysis due to lack of data. Among them, only 602 patients out of 1174 were evaluated pCR rates in the first interim analysis in Keynote-522 trail, [17], 7 patients did not proceed to surgery in I-SPY2 trial [18], and 6 patients withdrew consent in Nci 10,013 [22]. Among, 865 early TNBC patients received NACT plus ICIs and 692 early TNBC patients received NACT. The results showed that patients with TNBC who received NACT plus immunotherapy had a significant improvement in pCR compared with NACT alone in ITT populations (OR:1.91; 95% CI: 1.32–2.78, *P* < 0.001). Due to the large heterogeneity (I^2^ = 61%; *P* = 0.03), as shown in Fig. 2, the random effects model was used, and the funnel plot displayed a mild asymmetry. When eliminating the I-SPY2 Trial [18] (OR: 1.65; 95% CI: 1.23–2.20, *P* < 0.001), heterogeneity was greatly reduced (I^2^ = 37%; *P* = 0.18).

Due to the heterogeneity among RCTs, three subgroup analyses were performed according to the PD-L1 expression status, nodal status, and ECOG PS.

### Subgroup analysis

**Fig. 3:**
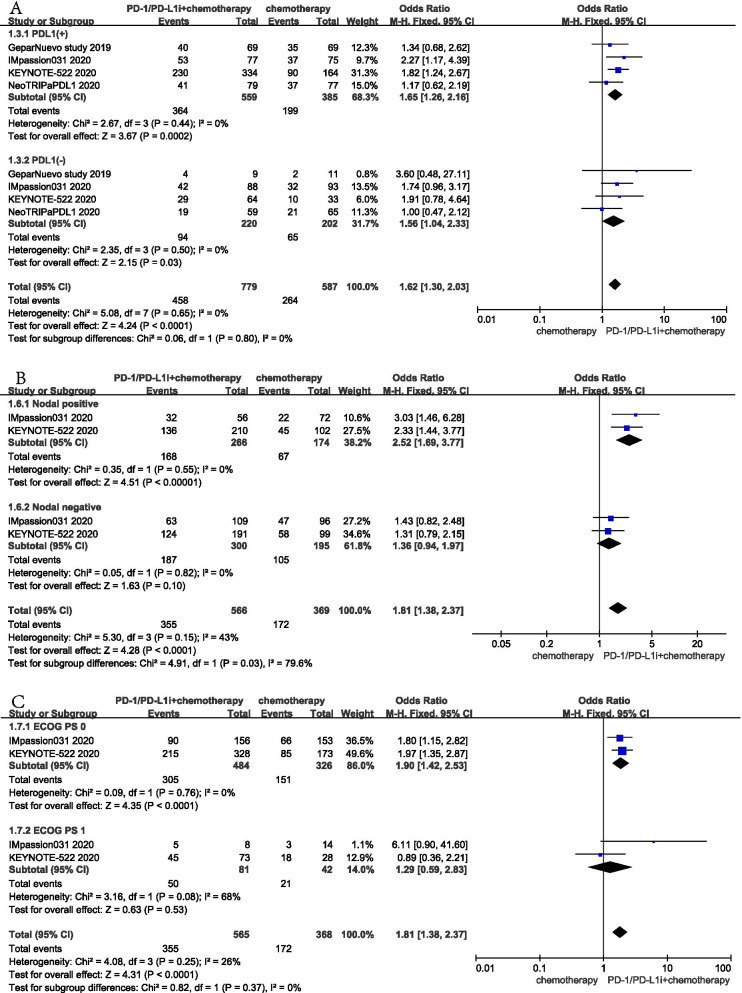
Pooled odds ratios for pathological complete response (pCR) of neoadjuvant chemotherapy plus immune checkpoint inhibitors versus neoadjuvant chemotherapy in three subgroups analysis. **A** The pCR analysis according to different programmed death ligand 1 expression status. **B** The pCR analysis according to different nodal status. **C** The pCR analysis according to different Eastern Cooperative Oncology Group performance-status score

According to the expression status of PD-L1, a total of 1366 patients participated in four studies to evaluate the efficacy of NACT plus ICIs [17, 19–21]. The results showed that the improvement in pCR rates was associated with PD-1/PD-L1 inhibitors, regardless of the expression status of PD-L1. Nevertheless, the benefit was significant in PD-L1 positive (OR: 1.65; 95% CI: 1.26–2.16; *P* < 0.001) compared with PD-L1 negative patients (OR: 1.56; 95% CI: 1.04–2.33; *P* = 0.03) with no heterogeneity (I^2^ = 0%, *P* = 0.65) Fig. 3A.

Subgroup analysis showed that NACT plus ICIs improved pCR rates in nodal positive patients (OR: 2.52; 95% CI: 1.69–3.77; *P* < 0.001) compared with NACT alone, but not in nodal negative patients (OR: 1.36; 95% CI: 0.94–1.97; *P =* 0.1) Fig. 3B. ECOG PS analysis showed that the ECOG PS 0 subgroup benefited from NACT plus ICIs compared with NACT alone (OR: 1.90; 95% CI: 1.42–2.53; *P* < 0.001), but not the ECOG PS 1 subgroup (OR: 1.29; 95% CI: 0.59–2.83; *P =* 0.53) Fig. 3C. This result showed that immunotherapy significantly improved pCR in patients with TNBC, especially in PD-L1 positive and high-risk populations (nodal positive). Furthermore, the physical status of patients (ECOG PS) was associated with an improvement in pCR.

### EFS rates analysis

**Fig. 4:**
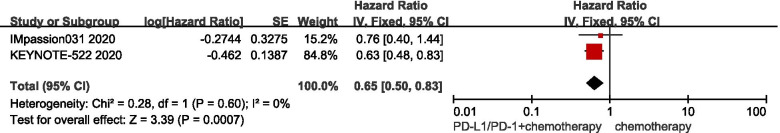
Pooled hazard ratios with 95% CI for event-free survival of neoadjuvant chemotherapy plus immune checkpoint inhibitors versus neoadjuvant chemotherapy in the overall populations

Only three of six studies (*N* = 1615) assessed EFS pooled hazard ratios (HRs) with 95% CI because of the lack of EFS data [17, 18, 20] (Fig. 4). However, the HR rates for EFS without 95% CI could be used to assess the I-SPY2 Trial [18]. According to the preliminary analysis of the other two studies, the results showed that NACT plus ICIs could increase EFS compared with NACT alone (HR: 0.65; 95% CI: 0.50–0.83; *P* = 0.0007) after 39.1 months of follow-up in the KEYNOTE-522 trial and 20.6 months in the IMpassion031 trial with no heterogeneity (I^2^ = 0%, *P* = 0.62). However, the data are immature and incomplete, and it is currently impossible to assess the EFS of NACT plus immunotherapy.

### Adverse events analysis

Safety analysis of 2205 patients with early TNBC after treatment in five eligible studies was conducted [17–21]. The results showed that NACT plus ICIs were associated with the incidence of toxicities, regardless of the grade of AEs (OR: 1.19; 95% CI: 1.05–1.34; *P* = 0.005), or grade 3–4 AEs (OR: 1.25; 95% CI: 1.08–1.45; *P* = 0.002). The addition of immune checkpoint inhibitors increased any grade of vomiting (OR: 1.37; 95% CI: 1.00–1.86; *P* = 0.05), hypothyroidism (OR: 5.04; 95% CI: 3.02–8.39; *P* < 0.001), hyperthyroidism (OR: 6.04; 95% CI: 2.39–15.29; *P* < 0.001) (eFigure 1 in the Supplement), and grade 3–4 diarrhea (OR: 2.54; 95% CI: 1.21–5.32; *P* = 0.01) compared with chemotherapy alone (eFigure 2 in the Supplement). Other side effects, regardless of chemotherapy-related or immune-related side effects, were not significantly different.

## Discussion

A total of six RCTs were included in our meta-analysis (*N* = 2142), which is the most comprehensive study with a larger sample size compare with previous meta-analysis to the best of our knowledge, aiming to evaluate the efficacy and safety of NACT plus ICIs vs. NACT alone in patients with early TNBC [17–22].The results showed that chemo-immunotherapy could improve pCR rates significantly compared with chemotherapy alone in patients with TNBC. Moreover, there was no correlation between the improvement in pCR rates and PD-L1 expression status; nevertheless, the pCR rates were higher in PD-L1 positive patients. Subgroup analysis showed that nodal positive and ECOG PS 0 subgroups benefited from PD-1/PD-L1 inhibitors plus NACT. Furthermore, a statistical difference was observed for EFS in the two RCTs through preliminary analysis. Finally, the addition of PD-1/PD-L1 inhibitors increased the incidence of grade 3–4 diarrhea, vomiting, hypothyroidism, and hyperthyroidism of any grade, did not increase the incidence of other adverse events.

Anthracyclines and taxanes are the most classic chemotherapy regimens for TNBC patients [25]. Platinum-based neoadjuvant chemotherapy can significantly improve pCR rates in patients with TNBC, but with large hematological toxicities [11, 26]. The effective rate of patients with TNBC still needs to be improved, and it is necessary to explore new antitumor drugs to increase efficacy. As immunotherapy is becoming increasingly effective in the treatment of cancer, it has become a promising treatment option for patients with TNBC [27–29]. Immunotherapy mainly uses the human immune system to kill tumor cells, thus achieving its anti-tumor effects, and aims to improve immunity [30, 31]. PD-L1 expressed by tumor cells binds to PD-1 expressed by T cells, thus inactivating T cells and forming immune escape of tumor cells, which can be blocked by PD-1 or PD-L1 inhibitors in the tumor microenvironment [31]. Due to the heterogeneity of the tumors, immunotherapy alone is not outstanding, and immunotherapy plus chemotherapy, target drugs, and local ablation therapy are expected to improve efficacy at present [32].

Based on the results of IMpassion 130 and Keynote-355 trials, atezolizumab and pembrolizumab in combination with chemotherapy have been shown to improve PFS in patients with advanced TNBC (aTNBC), especially PD-L1 positive patients [15, 16]. However, IMpassion131 assessed atezolizumab plus paclitaxel in aTNBC and the result showed did not improve progression-free survival (PFS) and overall survival (OS). The results of both studies were discussed and voted, in favor of continuing to approve atezolizumab in combination with nab-paclitaxel in patients with aTNBC of PD-L1 positive patients. However, recently the FDA stopped supporting this approval due to changes in the treatment outlook. In this situation, it is predicted whether patients with early TNBC will obtain significant benefits from immune checkpoint inhibitors. Some studies exploring the question, such as the Keynote-173 phase 1b study, included 60 high-risk patients with early stage TNBC in six cohorts to assess the safety and curative effect of adding pembrolizumab to six different regimens of chemotherapy [23]. All cohorts of the overall pCR were defined as ypT0/Tis ypN0, and the pCR rates was 60% (90% CI: 0.49–0.71). The EFS and OS at 12 months ranged from 80 to 100%.

In a previous meta-analysis [33], a total of three RCTs were included (*N* = 883) and the results showed that patients with TNBC had a pCR rate improvement after using immunotherapy plus chemotherapy (OR:1.78; 95% CI:1.34–2.30, *P*<0.0001) [16–18]. However, there may be high heterogeneity due to the lack of subgroup analysis. In addition, no survival results were reported in the previous meta-analysis. There could be some bias due to the small number of included studies and the small sample size. Another meta-analysis [34] included five RCTs; their sample size was smaller than ours (1496 vs 1557), and the result of the pCR rate analysis was consistent with ours (OR: 1.72; 95% CI: 1.22–2.42, *P <* 0.0001) [17–21]. Nevertheless, subgroup analysis only analyzed PD-L1 expression status and showed that PD-L1 negative populations did not benefit from NACT plus ICIs (OR: 1.56; 95% CI: 0.80–3.03), whereas we had a different result. Furthermore, the author did not analyze EFS by forest map combined analysis. In the analysis of AEs, their results showed that the addition of PD-1/PD-L1 inhibitors did not increase the incidence of AEs, while our AEs analysis showed different results. Therefore, it is necessary to perform a comprehensive meta-analysis to clarify and define the efficacy of immunotherapy and chemotherapy.

Some related studies have shown that pCR as a biomarker after neoadjuvant chemotherapy has a prognostic value in TNBC patients [35, 36]. It has been proven to be a substitute indicator for disease-free survival (DFS), EFS, and OS among patients with TNBC [37, 38]. There were six RCTs included in our meta-analysis (*N* = 2142) [17–22], and the results showed that immunotherapy combined with chemotherapy significantly improved pCR rates (OR: 1.91; 95% CI: 1.32–2.78, *P <* 0.001). The pCR rates were increased by 16.5% in IMpassion031 [20], 13.6% in KEYNOTE-522^17^, and 36.8% in Nci 10,013 [22]. However, the negative result in NeoTRIPaPDL1 [21] may be associated with the enrolled population being at high risk, the disease stage being late, and the immune induction effect of the chemotherapy regimens in the study design being insufficient.

However, our meta-analysis has some heterogeneity (I^2^ = 62%), and subgroup analysis is required. Some studies, such as IMpassion 130 and Keynote-355, showed that PD-L1 expression status is associated with better efficacy in patients with metastatic TNBC, but not in patients with early TNBC [15, 16]. The IMpassion 031, Keynote-522, and GeparNuevo studies showed benefits regardless of PD-L1 positivity or negativity in patients with early TNBC [17, 19, 20]. Moreover, the measurement of PD-L1 expression status was inconsistent between IMpassion 130 and Keynote-522. According to the PD-L1 staining of tumor-infiltrating immune cells as a percentage of the tumor area (PD-L1 negative < 1 vs. PD-L1 positive ≥1) in IMpassion 130, while calculating the combined positive score (CPS) to evaluate the number of PD-L1 positive cells (PD-L1 positive CPS ≥1) in Keynote-522 [20, 39]. In our meta-analysis, according to a subgroup analysis of PD-1/PD-L1 expression status, the results showed that PD-1/PD-L1 inhibitors plus chemotherapy had an improvement in pCR rates compared with chemotherapy alone, especially higher in patients with PD-L1 positive TNBC. The disease stage may indicate that different stages have different efficacy. The explanation is that the tumor immune microenvironment is stronger in early stage TNBC than in advanced-stage TNBC for PD-L1 patient populations; as a result, immunotherapy may further enhance the anti-tumor response. In addition, chemotherapy could stimulate some immune responses through the immune system [20]. Furthermore, two subgroup analyses showed that NACT plus ICIs increased pCR rates in nodal positive, ECOG PS 0 subgroups.

EFS was used to evaluate the efficacy of NACT. Three studies reported survival outcomes, and the results showed a statistical significance (HR: 0.66; 95% CI: 0.48–0.92, *P =* 0.01), although the data were immature. After 15.5 months of follow-up, KEYNOTE-522 showed that EFS was not achieved in the two groups at the time of the second interim analysis, however, after 39.1 months of follow-up, the result was beneficial. Among them, the I-SPY2 Trial showed that the addition of PD-1/PD-L1 inhibitors could improve EFS in patients with TNBC, but the IMpassion031 trial showed that EFS was not beneficial, possibly because of the short follow-up time [17, 18, 20].

According to our meta-analysis, NACT plus ICIs increased the incidence of diarrhea, vomiting, hypothyroidism, and hyperthyroidism and did not increase the incidence of other AEs compared with chemotherapy alone in patients with early TNBC. The intestinal tract as an immune organ, when attacked by the immunotherapy drugs, will cause some immune-related reactions, such as diarrhea [40, 41]. The highest incidence of AEs was neutropenia (349/1106 vs. 201/819), followed by anemia (165/1244 vs. 82/819), which was consistent with chemotherapy alone.

## Limitations

Our meta-analysis has some limitations. First, two of the included studies were published by abstracts, which would be associated with a greater risk of bias because the date is not comprehensive, and the research method and background are not clear. Second, there are some publication biases because of the limited number of studies and the small sample size. Third, different chemotherapy regimens among studies may influence the interpretation of the results. Finally, there are some difficulties in clarifying the survival outcomes of neoadjuvant immune checkpoint inhibitors plus chemotherapy due to only one study reporting OS; the data about EFS was immature and had a short follow-up time.

## Conclusions

This meta-analysis based on six RCTs showed that PD1/PD-L1 inhibitors plus chemotherapy have a higher pCR rate than chemotherapy alone in patients with early TNBC, especially in PD-L1 positive and high-risk populations with positive nodal disease, with an increased incidence of some immune-related AEs. This result further suggests that NACT plus ICIs might be an option in patients with in PD-L1 positive and high-risk populations with positive nodal disease early TNBC. However, the effect of immunotherapy on survival is still unclear. So, definite efficacy and accurate prognosis require further follow-up and analysis of predictive biomarkers.

## Supplementary Information

**Additional file 1: eTable 1.** The results of the studies included in the meta-analysis.

**Additional file 2: eFigure 1.** Any grade adverse events analysis in neoadjuvant chemotherapy plus immune checkpoint inhibitors versus neoadjuvant chemotherapy groups.

**Additional file 3: eFigure 2.** Grade 3-4 adverse events analysis in neoadjuvant chemotherapy plus immune checkpoint inhibitors versus neoadjuvant chemotherapy groups.

**Additional file 4: eFigure 3.** Risk of bias graph: review authors^,^ judgements about each risk of bias item presented as percentages across all included studies.

**Additional file 5: eFigure 4.** Risk of bias summary: review authors^,^ judgements about each risk of bias item for each included study.

## Data Availability

Not applicable.
