# Supplementary material for: Immune checkpoint inhibitors plus neoadjuvant chemotherapy in early triple-negative breast cancer: a systematic review and meta-analysis
Source: BMC Cancer. 2021 Nov 23;21:1261. doi: 10.1186/s12885-021-08997-w (PMC8609839; doi:10.1186/s12885-021-08997-w)
Supplement: Supplementary file 1 — Additional file 1: eTable 1. The results of the studies included in the meta-analysis. [file 12885_2021_8997_MOESM1_ESM.doc]

| eTable 1. The results of the studies included in the meta-analysis. | | | | | | | | |  |
| --- | --- | --- | --- | --- | --- | --- | --- | --- | --- |
| study | No. of  patients | pCR increased(↑) in ITT | pCR (↑) in PD-L1(+) | pCR (↑) in PD-L1(-) | pCR(↑) in nodal (+) | pCR(↑) in nodal (-) | pCR(↑) in ECOG PS 0 | pCR(↑) in ECOG PS 1 | HR for EFS |
| KEYNOTE-522 | 602 | +13.6% in EA | +14.0% in EA | +15.0% in EA | +20.7% in EA | +6.3% in EA | +16.4% in EA | -2.7% in EA | 0.63(95%CI,0.43-0.93) |
| GeparNuevo | 174 | +9.2% in EA | +7.3% in EA | +26.2% in EA | NA | NA | NA | NA | NA |
| NeoTRIPaPDL1 | 280 | +2.7% in EA | +3.9% in EA | 0 in EA | NA | NA | NA | NA | NA |
| I-SPY2 Trial | 107 | +38.0% in EA | NA | NA | NA | NA | NA | NA | 0.60(95%CI,NA) |
| Impassion031 | 333 | +16.5% in EA | +19.5% in EA | +13.3% in EA | +27% in EA | +9% in EA | +15% in EA | +41% in EA | 0.76(95%CI,0.40-1.44) |
| Nci 10013 | 61 | +36.8% in EA | NA | NA | NA | NA | NA | NA | NA |
| EA:Experimental arm; NA:not available; ECOG PS:Eastern Cooperative Oncology Group performance-status score; pCR:pathological complete response; PD-L1+:programmed death ligand 1 positive; PD-L1-:programmed death ligand 1 negative | | | | | | | | | |
