# Supplementary figures and images for: Immune checkpoint inhibitors plus neoadjuvant chemotherapy in early triple-negative breast cancer: a systematic review and meta-analysis
Source: BMC Cancer. 2021 Nov 23;21:1261. doi: 10.1186/s12885-021-08997-w (PMC8609839; doi:10.1186/s12885-021-08997-w)

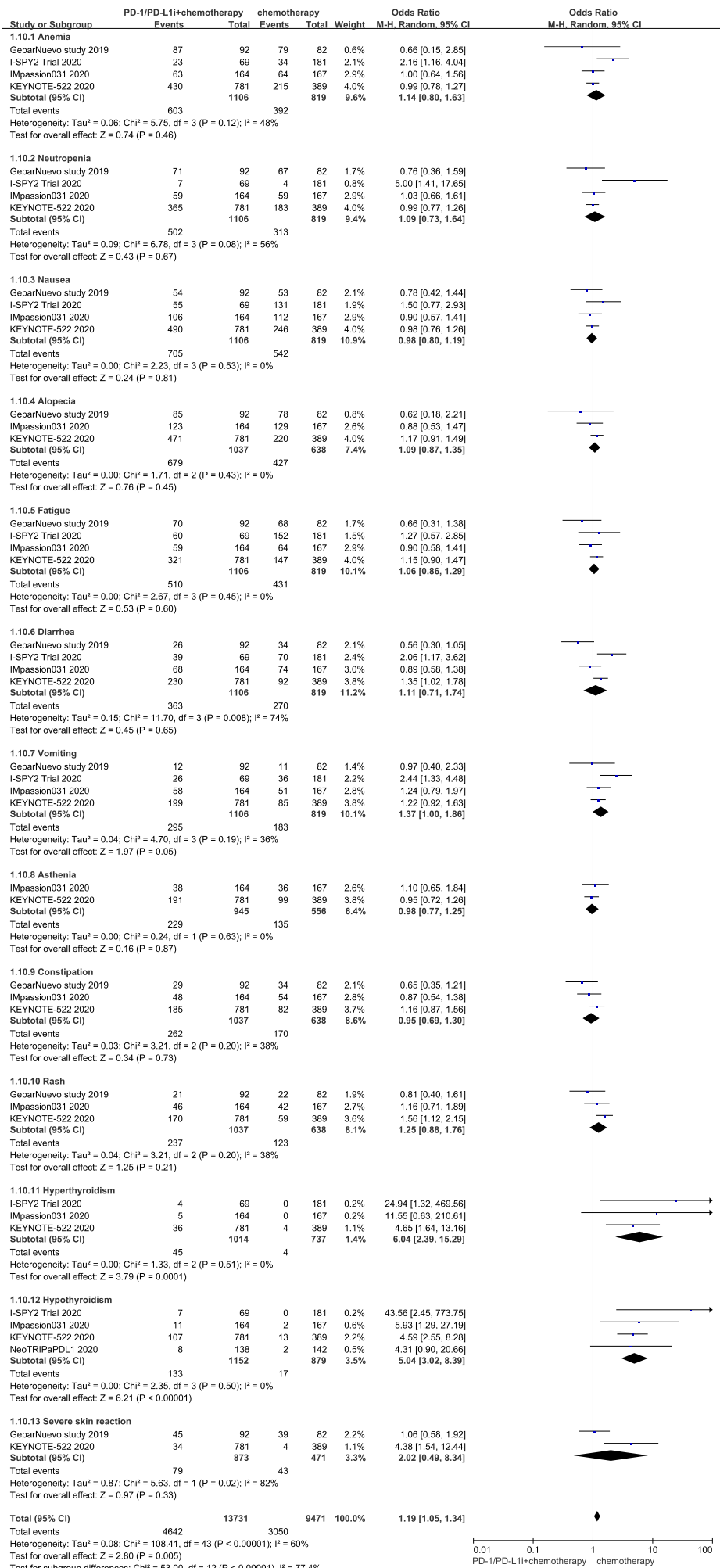

Supplement: Supplementary file 2 — Additional file 2: eFigure 1. Any grade adverse events analysis in neoadjuvant chemotherapy plus immune checkpoint inhibitors versus neoadjuvant chemotherapy groups. [file 12885_2021_8997_MOESM2_ESM.pdf]

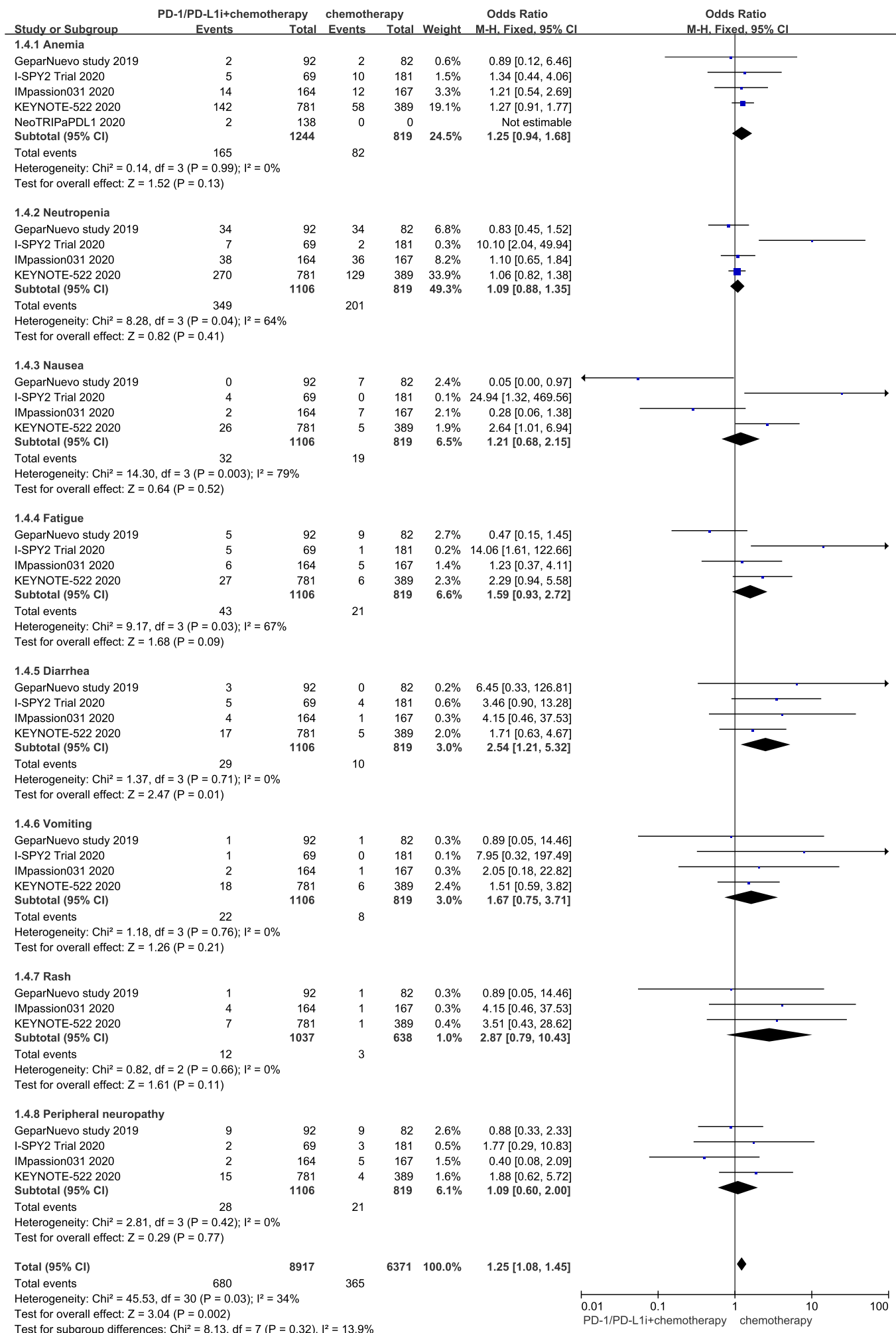

eFigure 2.

Supplement: Supplementary file 3 — Additional file 3: eFigure 2. Grade 3-4 adverse events analysis in neoadjuvant chemotherapy plus immune checkpoint inhibitors versus neoadjuvant chemotherapy groups. [file 12885_2021_8997_MOESM3_ESM.pdf]

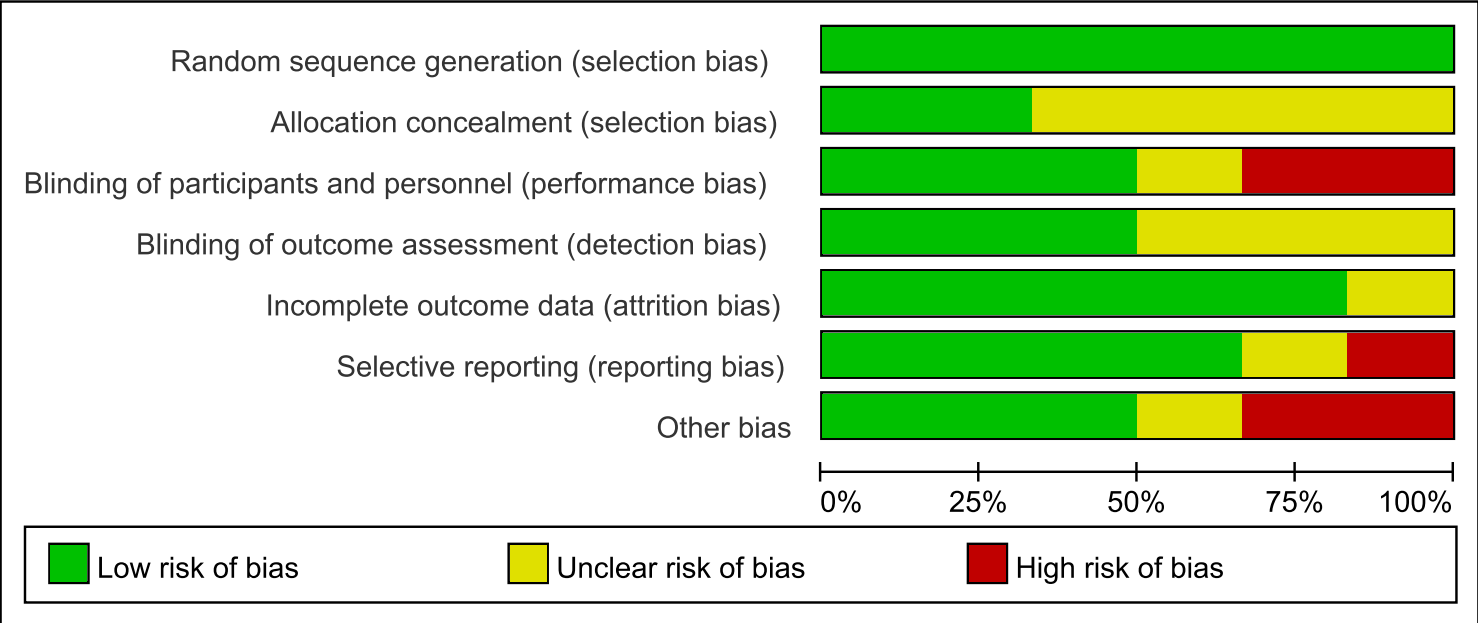

eFigure 3.

Supplement: Supplementary file 4 — Additional file 4: eFigure 3. Risk of bias graph: review authors, judgements about each risk of bias item presented as percentages across all included studies. [file 12885_2021_8997_MOESM4_ESM.pdf]
