# Supplementary material for: Immune checkpoint inhibitors plus neoadjuvant chemotherapy in early triple-negative breast cancer: a systematic review and meta-analysis
Source: BMC Cancer. 2021 Nov 23;21:1261. doi: 10.1186/s12885-021-08997-w (PMC8609839; doi:10.1186/s12885-021-08997-w)
Supplement: Supplementary file 5 — Additional file 5: eFigure 4. Risk of bias summary: review authors, judgements about each risk of bias item for each included study. [file 12885_2021_8997_MOESM5_ESM.pdf]

| | Random sequence generation (selection bias) | Allocation concealment (selection bias) | Blinding of participants and personnel (performance bias) | Blinding of outcome assessment (detection bias) | Incomplete outcome data (attrition bias) | Selective reporting (reporting bias) | Other bias |
|---|---|---|---|---|---|---|---|
| GeparNuevo study 2019 | + | ? | + | + | + | + | + |
| IMpassion031 2020 | + | + | + | + | + | + | + |
| I-SPY2 Trial 2020 | + | ? | − | + | + | − | − |
| KEYNOTE-522 2020 | + | + | + | ? | + | + | + |
| Nci 10013 2020 | + | ? | ? | ? | + | + | − |
| NeoTRIPaPDL1 2020 | + | ? | − | ? | ? | ? | ? |

eFigure 4.
